# Bat-associated microbes: Opportunities and perils, an overview

**DOI:** 10.1016/j.heliyon.2023.e22351

**Published:** 2023-11-18

**Authors:** J. Dhivahar, Anutthaman Parthasarathy, Kathiravan Krishnan, Basavaraj S. Kovi, Ganesh N. Pandian

**Affiliations:** a Research Department of Zoology, St. Johns College, Palayamkottai, 627002, India; b Department of Plant Biology and Biotechnology, Laboratory of Microbial Ecology, Loyola College, Chennai, 600034, India; c Department of Biotechnology, Laboratory of Virology, University of Madras, Chennai, 600025, India; d Department of Chemistry and Biosciences, Richmond Building, University of Bradford, Bradford, West Yorkshire, BD7 1DP, United Kingdom; e Institute for Integrated Cell-Material Sciences (WPI-iCeMS), Yoshida Ushinomiyacho, 69, Sakyo Ward, 606-8501, Kyoto, Japan

**Keywords:** Bats, Zoonotic disease, Bat seeding, *Borrelia*, *Bartonella*, *Trypanosoma cruzi*, *Plasmodium*, Coronavirus, SARS, MERS, SARS-CoV-2

## Abstract

The potential biotechnological uses of bat-associated bacteria are discussed briefly, indicating avenues for biotechnological applications of bat-associated microbes. The uniqueness of bats in terms of their lifestyle, genomes and molecular immunology may predispose bats to act as disease reservoirs. Molecular phylogenetic analysis has shown several instances of bats harbouring the ancestral lineages of bacterial (*Bartonella*), protozoal (*Plasmodium*, *Trypanosoma cruzi*) and viral (SARS-CoV2) pathogens infecting humans. Along with the transmission of viruses from bats, we also discuss the potential roles of bat-associated bacteria, fungi, and protozoan parasites in emerging diseases. Current evidence suggests that environmental changes and interactions between wildlife, livestock, and humans contribute to the spill-over of infectious agents from bats to other hosts. Domestic animals including livestock may act as intermediate amplifying hosts for bat-origin pathogens to transmit to humans. An increasing number of studies investigating bat pathogen diversity and infection dynamics have been published. However, whether or how these infectious agents are transmitted both within bat populations and to other hosts, including humans, often remains unknown. Metagenomic approaches are uncovering the dynamics and distribution of potential pathogens in bat microbiomes, which might improve the understanding of disease emergence and transmission. Here, we summarize the current knowledge on bat zoonoses of public health concern and flag the gaps in the knowledge to enable further research and allocation of resources for tackling future outbreaks.

## 1 Introduction

The still ongoing Covid-19 pandemic has brought the issues of infectious diseases, especially those of zoonotic origins to the fore. The pandemic emerged because of several overlapping global trends such as climate change, deforestation, urbanisation, human population expansion into formerly less disturbed areas and the increased ease of domestic and international travel. In this context, scientific interest in zoonotic diseases is increasing, and the growth of high throughput and deep sequencing technologies can compensate for the important limitations of culture-based approaches. Recent work relevant to zoonosis includes methods for improved taxonomic classification of uncharted sequences [1], databases for animal-associated microbes such as the AMDB database for non-human animal gut microbiomes [2] and large-scale efforts to extract and sequence bacterial genomes from the guts of wild animals [3]. Metagenomics approaches are capable of distinguishing between pathogens and non-pathogens of the same strain differing only in unknown single genes [4], and therefore suited to analysing emerging pathogens from complex and rare samples, for which specific analytical methods may or may not be available. Moreover, speed, cost and sample size criteria are significantly better than for culture-based approaches. Other advantages include the potential to track pathogens from different kingdoms plus viruses simultaneously from the same samples bypassing specialized cultivation or cell-infection experiments. Characterising the microbiota of wildlife in this way will identify the natural reservoir animals of pathogens, paving the way for tracking pathogen dissemination to humans and providing early warnings of possible outbreaks [3].

Bats are one of the important taxa of urban-adapted wildlife worldwide and were originally thought to be the source of the Covid-19 strains, but it is now considered that Covid-19 is a mosaic of ancestral Rhinolophus bat and pangolin strains [5]. They have been linked to the epidemiology of not only Covid-19, but also various other diseases [6,7] and are considered as significant reservoirs and carriers of emerging infectious diseases [8]. Some of the zoonoses are directly transmitted from bats to humans, whereas others are descendants of pathogens occurring ancestrally in bats. In addition to the direct public health interest in bats, they have been proposed as model systems for microbiome research in wildlife to learn about metagenomes in host ecology and evolution [9]. Bats (Chiroptera) are the only type of mammal capable of powered flight and account for the second highest number of mammalian species, after the order Rodentia [10,11]. The current total diversity of bats is up to 20 % of all known extant mammal species globally, with over 1400 species [10,11]. Bats are found in the fossil record with fossils from 32 million years ago resembling modern bats [12], while the two oldest bat fossils date to about 48 [13] and 51 million years ago [14], respectively.

**Fig. 1:**
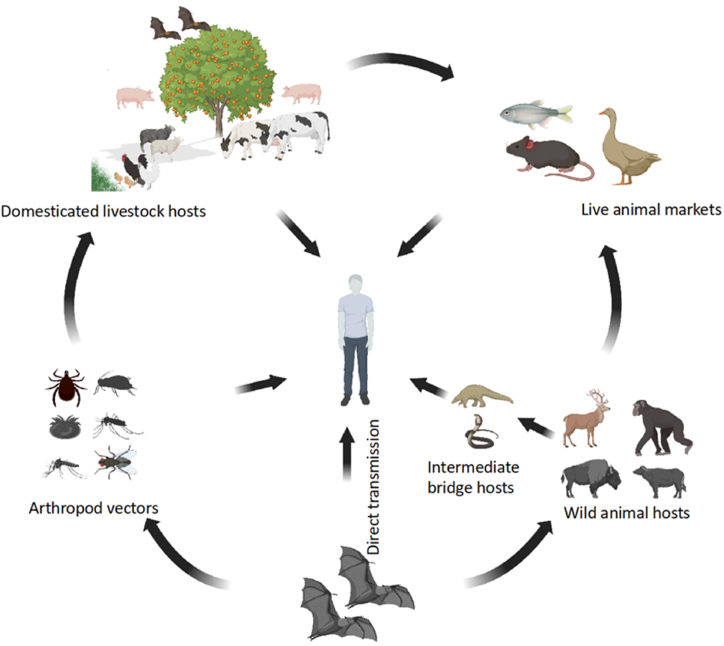
Routes of transmission of bat pathogens to humans in the ecosystem.

Bats have successfully occupied diverse ecological niches [15,16]. They perform a range of ecosystem services such as pollination, seed scattering, and insect-pest control [17]. Further, bat-associated microbes could be of biotechnological interest, since they harbour bacteria expressing enzymes such as cellulases, xylanases [18,19], amylases [20] and chitinases [20,21] as well as bacteria producing antifungals [21]. In several ways, bats may be considered the perfect host for emerging zoonotic pathogens. Some bats live in caves, and in these areas, they occur habitually in dense colonies/roosts. In addition, some also forage over wide areas, and thereby encounter other species of bats, other mammals, and arthropods (Fig. 1). Long flight distances have been recorded, in one instance, more than 2200 km [22]. While migration is known, there is a paucity of data on whether or how they can disseminate viruses, bacteria, fungi, or protozoan parasites over considerable distances. Bats’ exceptional immunity to most of the diseases they transmit has been attributed to the loss of genes for the pro-inflammatory regulator NF-κB, and augmented APOBEC3 [23], which encode immune functions, particularly antiviral functions [24,25].

Further, bat ectoparasites such as ticks may bite not only bats, but also carnivores, ungulates, and primates such as humans [26]. Therefore, the interactions of both bats and bat-biting vectors with other species are crucial to understand the emergence and transmission of these diseases. We support calls to avoid casting bats solely ss public health villains [27] since they also perform invaluable ecosystem services such as consuming insect pests and pollination, and instead call for also examining the role of human disturbances because disturbed habitats could prime bats for disease emergence [13]. In fact, for most zoonotic diseases, focussing only on the pathogen or the primary host species is inadequate, and for understanding disease emergence, persistence and transmission, ecosystem perspectives are essential [28]. In this review, we offer an overview of the accessible literature on the potential biotechnological applications of bat-associated bacteria and the potential risks of bat-associated pathogens (viruses, bacteria, protozoa, and fungi) covering bat genome/microbiome and ecological aspects.

### 1.1 The mammalian gut microbiome and bats

In most mammals, the gut is colonized by copious numbers of microorganisms, collectively called the gut microbiota, which is vital for host health [29,30]. The mammalian intestine contains a complex, dynamic, and spatially diversified society of non-pathogenic bacteria. The assortment of broad habitats and the convolution of the mammalian digestive tract contribute to a rich variety of commensal microbes with specific symbiotic associations. In mammals, gut microbiomes influence infections through the effects they exert on host physiology, immunity, nutrition, and behaviour. The robustness of the microbe-host system is based on a set of molecular interactions among the symbiotic partners [29,30]. Third-generation sequencing methods are improving the resolution of the taxonomic data, not only characterizing the community composition at the phylum and family levels, but increasingly also the species level, permitting better analysis of functions associated with shifts correlated to health and disease [31].

Ley et al. [32], found that in general, taxonomically closely related animals had analogous gut microbial compositions. In mammals, compositions of the gut microbial communities from different species with analogous diets appeared to congregate - herbivores, carnivores and omnivores have more similar gut microbiome compositions [33] despite differences in host phylogeny. For bats overall, host phylogeny, life history, physiology and geography have overlapping influences on microbiomes [32,34,35]. Further, the gut microbial communities can be prejudiced by the host's diet, metabolic and immune system development, chemical exposures, and initial colonizers despite their host specificity [36]. Additionally, host transcriptomics may shift in tune with the diurnal rhythms of the resident microbiota [37]. The gut microbial community may be affected by additional factors, such as captivity, which in bats causes the taxonomic and functional convergence of the gut microbiota [38].

The microbiome is shaped by the diet substrates that differentially support or augment the growth of specific microbes. This means that high variation in specific gut microbial taxa can be induced by specialized diets; for example, giant pandas (*Ailuropoda melanoleuca*) and red pandas (*Ailurus fulgens*) eat bamboo diets rich in fibrous material. When compared to the gut microbiomes of David's deer (*Elaphurus davidianus*), humans (*Homo sapiens*), cheetah (*Acinonyx jubatus*), black-backed jackal (*Canis mesomelas*), and black bear (*Ursus thibetanus*), giant panda gut microbiomes have a high degree of variation with respect to the *Pseudomonadaceae* and C*lostridiaceae*; this divergence may be due to unstable gut colonization, perturbation, or selective pressure due to their carnivorous gastrointestinal system [39]. This type of divergence based on phylogeny and feeding habits is also known in bats, whose gut microbiome resembles those of the Carnivora [40].

In the New World family of the Phyllostomidae (leaf-nosed bats), the gut microbiota of bats with divergent feeding habits were analysed: *Macrotus waterhousii* (insectivore), *Desmodus rotundus* (sanguivore or vampire), *Leptonycteris yerbabuenae* (nectarivore), *Glossophaga soricina* (nectarivore), *Carollia perspicillata* (frugivore) and *Artibeus jamaicensis* (frugivore) [41]. Overall, the microbiome correlated with phylogeny of the host and feeding habits, but plant-based diets led to lower overall diversity of the microbiome, while the vampire and insectivore strategies led to the most diverse microbiota [41]. The former also had less specificity, while the latter had more a clustered arrangement of the microbiome [41]. However, bat microbiomes appear to have several unique features not found in non-flying mammals.

## 2 The uniqueness of bat gut microbiomes

In mammals, gut microbiomes influence infections through the effects they exert on host physiology, immunity, nutrition, and behaviour. Bat gut microbiomes more closely resemble the Proteobacteria-dominated gut microbiomes of birds than those of other mammals [42]. In particular, the anaerobic phylum Bacteriodetes, which is common in the guts of terrestrial mammals, is rare in bat guts [40]. As an adaptation to flight, bats have rapid gut transit times which may reduce microbiome stability, constrain nutrient uptake, and affect pathogen exposure and the evolution of tolerance mechanisms. Experimental and longitudinal studies are needed to understand the function of bats gut microbiomes and their role in modulating viral infection dynamics [42].

The enrichment of Proteobacteria in human guts signals dysbiosis of the microbiota, and several Enterobacteriaceae are potentially opportunistic human pathogens [43,44]. Furthermore, the gut microbiome composition found in bats if present in humans would cause various inflammatory diseases in humans but bats themselves seem to suffer no major repercussions from hosting such a microbiome [45]. Suppression of the NOD-like receptor protein-3 (NLRP3) inflammasome is related to the gut microbiome's composition and biochemical functions [46,47] and therefore, the viral tolerance of bats may be due to their unique gut microbiomes [48]. Due to these reasons, bacterial and viral colonization of bats may be asymptomatic, but when transmitted to humans, these microbes may cause diseases. Another striking aspect of bat gut physiology is that evolutionary history and diet seem only weakly correlated to microbiome composition (as in birds), in contrast to nonflying mammals, presumably due to the physiological demands imposed by flight [49].

### 2.1 Bat microbiomes and zoonotic diseases

Increasing knowledge of bat microbiomes can further the study of their vital roles in the ecosystem, help explain the potential ecological consequences of habitat destruction and also promote an understanding of bat zoonotic diseases. Federici et al., have stated that several pathogenic bacteria including multidrug resistant strains are “common guests” of bats and have therefore emphasised prevention of bat habitat destruction to minimise zoonotic transmission [50]. They have also compiled maps of bacterial and eukaryotic genera colonising the various body parts of bats. There are many studies which look at potential or confirmed pathogens from bat samples. However, the pathogenic potential of all these bat-associated strains has not been assessed and vital information about possible transmission among bats and further to humans, domestic animals, and common wildlife is missing. This is a critical knowledge gap, and the implications for human and veterinary medicine needs further study [51]. This gap can to a large extent be addressed by metagenomics approaches examining bat microbiome composition.

Several recent studies have used the metagenomics of the 16S rRNA gene to identify potential bacterial pathogens in bats in a culture-independent fashion [3,[52], [53], [54]]. Amplicon sequencing for bat species level comparisons and amplicon plus shotgun sequencing for colony level comparisons might be beneficial to discern the functions of members of the microbiome [54]. Most bats including fish-eating bats have gut microbiomes dominated by Proteobacteria, but fish-eaters harbour distinct (and possibly pathogenic) genera among the Proteobacteria such as *Chlamydia*, *Microcystis* and *Aeromonas*, which are rare or not found in insectivorous bats [54]. Metagenomics approaches using the 18S rRNA for eukaryotes identified the Ascomycota and Basidiomycota as the dominant fungi in the guts of Chinese bats [55], and showed that herbivorous bats carried more fungi, many of which are foodborne pathogens in humans also.

Metagenomic studies of the bat virome have also been reported. For example, a Swiss study detected 39 virus families, of which 16 could possibly infect vertebrates, from the faeces deposited by and tissue samples from 18 European bat species [56]. This study showed that fallen stools of bats could be tracked by metagenomic surveys to assess possible zoonosis. Additionally, the monitoring of oral and gut virome diversity in bats, birds, dogs, and cats has also been proposed to uncover novel viruses capable of human infections [57]. A high identity between bat-associated viral contigs and human-associated ones would be suggestive of crossing the metazoan species barrier, as has been observed for influenza A and coronavirus strains in China [58]. Lestari et al., sequenced rotavirus G3 strains from human samples of diarrhoeal episodes in Bangkok and aligned the deduced amino acid sequences, which showed an unusual G3-P10 combination resembling those of bat strains [59]. Even though rotaviruses often undergo recombination to generate new strains, they concluded that the atypical G3-P10 sequence suggested transmission from bats to humans in urban areas. Thus, using sequence information from human samples or wastewater might also enable the surveillance of bat zoonosis in cases where the bat-related sequences are already known.

Metagenomics also showed that the composition of bacteria in the bat gut differs between the large intestine, the small intestine, and the faeces, meaning that faecal analysis cannot be a good proxy for community composition in different bat gut compartments [60]. Like most other animals, bats carry distinct microbiomes in different body niches. This includes the excreted microbiome as well, whereby the saliva had a very high dominant proportion of Proteobacterial genera (the Pasteurellaceae and Neisseriaceae families were enriched), compared to the urine and faeces in insectivorous bats [61]. The urine was more diverse and enriched in *Leptospira*, while *Bartonella* was abundant in faeces, and *Bartonella*, *Burkholderia*, and *Helicobacter* were found in urine, faeces, and saliva [61]. Thus, the identity of the suspected/confirmed human pathogen depends on the excreta type, which has implications for disease transmission to humans. Another study using PacBio sequencing in the faeces of Chinese bats identified 480 species-level phylotypes (SLP), of which 32 were known human pathogens, while another 40 were flagged as potential human pathogens [62]. Monitoring of bacterial metagenomes shed in bats’ saliva, urine, and faeces may enable the surveillance and early detection of zoonotic pathogens.

An exciting and surprising finding from a recent study by Popov et al., is that bats' gut bacterial community is involved in DNA damage as well as antioxidant activity [63]. Lactobacilli and bacilli isolated from bat faeces could elevate the expression of the genes *recA* and *katG*, the first of these induces DNA strand breaks, while the second usually detoxifies hydroxyl radicals [63]. Thus, the gut bacterial community plays a role in bats’ antiviral response, which might help develop strategies to prevent the transmission of bat-borne viruses. Wasimuddin et al., found that shifts in the gut bacterial community composition of bats are correlated with astrovirus infections [64]. Astroviral infection in both adult and juvenile bats resulted in microbiome shifts termed “pathobiont-like”, characterised by elevated levels of *Haemophilus*, *Mycoplasma*, and *Streptococcus*, as well as antagonistic interactions between commensals and possible pathogens. Antagonism between suspected pathogenic Streptococcus and Xanthomonadaceae, as well as Peptostreptococcaceae and Enterobacteriaceae was reported in the bat gut community [64].

Bats have Proteobacteria-dominated gut communities and therefore the abundance of the members of this phylum shifts in ways contrary to trends in humans. However, the abundance of Firmicutes and Actinobacteria falls post-astroviral infection in young bats but increases in adult bats. Both these phyla are presumably involved in colonization resistance in bats, which is better developed in adults than juveniles (which may lose core taxa due to ongoing astroviral infection). This has parallels in humans: children suffer from diarrhoea following the loss of core taxa while adult humans have less severe reactions [65]. In the human and mouse microbiomes, these taxa relate to immune-modulated colonization resistance [66]. These types of core taxa vs. pathogen interactions and taxa abundance shifts linked to dysbiotic states will help monitor the emergence and transmission dynamics of not only bacterial but also viral diseases of bats having public health importance.

Linking taxonomy and ecological factors might also be a good way to understand the emergence of bat zoonoses correlated with microbiome shifts and identify bat populations most likely to transmit to humans. An elegant metabarcoding study by Mohd-Yusof et al., using the 16S rRNA gene recently conducted in four island populations of the same bat species, the frugivorous flying fox *Pteropus hypomelanus* [67]. Higher gut bacterial diversity was observed in Redang and Langkawi islands, compared to Tinggi and Pangkor islands. The former two were better habitats than the latter two, which had more human disturbances, leading to a lower quality of bat diet and hence, altered microbiomes. Genus-level abundance of potential pathogens such as *Micrococcus*, *Pseudoalteromonas*, and *Chryseobacterium* increased in the Redang samples, while *Klebsiella*, *Raoultella*, *Enterobacter* and *Streptococcus* abundance was elevated in the Tinggi samples and the Pangkor samples showed increased abundances of *Paeniclostridium* and *Acinetobacter*.

### 2.2 Gut bacterial diversity in bat fauna

a) **Frugivorous bats:** Frugivorous bats include the Pteropodid bats in the Old World and the Phyllostomid bats in the Americas, which are only distantly related. They eat a wide range of flowers, fruits, and leaves to meet their nutritional energy demand [68]. Increasing awareness that a symbiotic interaction between resident bacteria and their host plays critical roles in the biology of both partners has stimulated research into the mechanisms of regulation of mutually beneficial non-pathogenic microbe-host interactions. This is an important question since the mammalian gut contains an incredible profusion of microbes that biosynthesize distinctive metabolites, some of which may have industrial potential. Gut microbial (particularly bacterial) enzymes and proteins are under intensive research due to their potential in food, animal feed, pulp and paper, textiles, and bio-fuel production [69].

Bats power their highly energetically demanding flying lifestyle by maintaining a high rate of carbohydrate utilization and nutrients are often directly and immediately burned to sustain high, abrupt energy needs for flight [70]. In fruit bats, the protein requirements are satisfied by ingesting pollen and leaves [71,72]. Cellulose and xylan are the major constituents of leaves, which must be broken down to access other nutrients. Animals commonly consume cellulose and xylan, but they do not produce cellulases and xylanases themselves, depending instead on a symbiotic affiliation with cellulolytic and xylanolytic microorganisms; fruit bats also harbour xylanolytic and cellulolytic bacteria [73]. Table 1 portrays the details of bacteria isolated from frugivorous bats. In recent times, xylanase-containing bacteria isolated from the Indian flying fox (*Pteropus giganteus*) faeces have been successfully applied in poultry feed pre-digestion [19] and the same xylanase enzyme was loaded on nanoparticles and used as a reductant in fruit juice clarification [74]. Previously, hyper-xylanase producing *Bacillus* spp. were isolated from *P. giganteus* faeces [18]. Xylanases and cellulases are of commercial value in utilizing food and agricultural wastes and since cells of leaves have 9–25 % cellulose and 20–50 % xylan [18], fruit bat guts might be a promising source to find these useful enzymes..

b) **Insectivorous bats:** Generally, in insectivorous bat metabolism, chitinase plays a vital role in digesting the chitin from the exoskeleton of insects consumed. Chitinases have attracted attention owing to their range of applications in the medical, agricultural, and food biotech fields. Microbes, chiefly bacteria, form one of the significant sources of chitinases, and chito-oligomers, N-acetyl D-glucosamines and biocontrol agents produced by chitinase catalysis are considered valuable [75]. Table 2 summarizes insectivorous bat species, bacteria, and potential industrial applications of the bacteria compiled from previous studies.
c) **Vampire bats:** The common vampire bat (*Desmodus rotundus*) microbiota is composed predominantly of the Phyla, *Firmicutes, Proteobacteria, Tenericutes*, and *Epsilonbacteraeota.* Vampire bats have gut microbiota that is compositionally discrete from those of other bats, which is expected due to their unique blood meals which cause a shortage of vitamins and lipids, as well as high nitrogen waste and challenges to osmotic homeostasis [76]. Most of the data comes from *D. rotundus* and not much is known about the microbiomes of other vampire bats. Not much is known about the microbiomes of other vampire bats.

**Table 1:** Bacterial diversity in frugivorous bats.

Family and species of bat	Country of origin	Source of isolation	Bacteria isolated	References
*C. tittaecheilus tittaecheilus*	Indonesia	Rectum	*Citrobacter* sp., *Enterobacter* sp., *Escherichia coli, Klebsiella* sp.	Graves et al. (1988) [77]
*C. brachyotis javanicus*			*Citrobacter* sp., *E. coli*	
*Cynopterus sphinx angulatus*			*Citrobacter* sp., *Enterobacter* sp., *E. coli, Klebsiella* sp.*, Pseudomonas* sp., *Serratia* sp.	
*Pteropus giganteus* *P. hypomelanosis*	Indonesia	Rectum	*Clostridium septicum, Micrococcus* sp. *and Staphylococcus* sp.	Heard et al. (1997) [78]
*P. pumilus* *Ptenochirus jagori* *C. brachyotis,* *Eonycteris spelaea, Rousettus amplexicaudatus*	Philippines	Rectum	*Corynebacterium* sp., *Proteus* sp., *Staphylococcus* sp., *Salmonella* spp.	
*P. giganteus*	India	Intestine	*Proteus vulgaris, Proteus mirabilis, Citrobacter freundii, Serratia liquefaciens, Klebsiella oxytoca*	Prem Anand and Sripathi (2004) [79]
*C. sphinx*			*Bacillus* sp.*, Clostridium* sp., *Streptococcus* sp., *Staphylococcus* sp.	Prem Anand et al. (2012) [73]
*C. brachyotis brachyotis*	Peninsular Malaysia	Stomach, Intestine	*B. cereus, Enterobacter* sp., *P. aeruginosa, Klebsiella oxytoca, Klebsiella pneumoniae, Bacillus thuringiensis, E. coli, E. hermannii, Serratia marcescens.*	Daniel et al. (2013) [80]
*P. giganteus*	India	Faeces	*B. amyloliquefaciens,* *B. methylotrophicus, B. velezensis, Pseudomonas nitroreductase*	Dhivahar et al. (2019 & 2020) [18,19,74]

**Table 2:** Bacteria with biotech potential and disease interest in insectivorous bats.

Family and species of bat	Enzyme/property	Source of isolation	Bacteria isolated	References
M. septentrionalis, M. lucifugus, M. sodalis, Eptesicus fuscus, P. subflavus, N. humeralis, L. borealis, L. cinereus, L. noctivagans	Chitinase	Stomach, Intestine	Hafnia alvei, Citrobacter amelonaticus, Enterobacter aerogenes, E. cloacae	Whitaker et al. 2004 [81]
Insectivorous bats	ESBL-producing Enterbacteriaecae	Faeces	*E. coli, K. pneumonia, Enterobacter cloacae*	Nguema et al. 2020 [82]
*Rhinopoma hardwickii, Megaderma lyra, Hipposiderous fulvus*	Bacterial classification	Guts	*Bacillus* sp., *Citrobacter* sp., *Enterobacter* sp	Gracy Jenifer et al. 2021 [83]
Insectivorous bats guano	Antifungal		Chitinolytic bacteria	Rahmawati et al. 2016 [21] Delfini et al. 2021 [84]
*Rhinolophus euryale*	Amylase, chitinase		*Colletotrichum* sp	Maxinova et al. 2017 [20]

### 2.3 Are bats reservoirs for bacterial pathogens?

Preceding studies have assessed the gastrointestinal bacteria in bats and reported that some strains are potentially pathogenic to humans [51]. In both *R. luctus* and *M. leucogaster*, the governing phylum was Proteobacteria (stomach 86.07 and 95.79 %, intestines 91.87 % and 88.78 %, respectively), followed by Firmicutes (stomach 13.84 and 04.19 % intestines, respectively). Overall, 18 and 20 bacterial genera occurred with a comparative abundance of 0.01 % or more in the gastrointestinal tracts of *R. luctus* and *M. leucogaster*, respectively. In *R. luctus,* the main genera were *Lactococcus* (10.11 %) and *Paeniclostridium* (3.41 %) in the stomach and *Undibacterium* (28.56 %) and *Paeniclostridium* in the intestines. In *M. leucogaster*, the governing genera were *Undibacterium* (54.41 %) and *Burkholderia* in the stomach, and *Undibacterium* (29.67 %) and *Enterococcus* in the intestines [85]. The microbiota may be monitored as an early warning sign of potential downstream effects on host ecology and fitness [86,87], since the comparative abundances of some core microbiota members are correlated to the innate immune status of the bat host [88].

Certain behavioural traits of bats may play a role in the transmission of bacterial diseases, both within the bat order and to other mammals. Aggregation in large roosts, movements between roosts, and migrations may facilitate the exchange of bacteria between different bat populations [89]. Bats also hibernate and during this process, their immune response could be suppressed [90], which may lead to disease when aroused [91]. While foraging, many bats encounter humans and domestic animals [92]. Whereas cats and dogs may attack and infect bats with bacterial pathogens, insectivorous bats may acquire pathogens from their prey [93]. Common human and veterinary pathogens from the genera *Salmonella*, *Yersinia* and *Campylobacter* may be acquired in this manner [[94], [95], [96]]. Contaminated fruits and water may also lead to infections in bats [92,95,96].

Most of the research to date has examined viral agents rather than the impact of bacteria in bat disease and mortality. Enteric pathogens in bats are thought to originate from their diet and foraging habitats, which in turn relates to the transmission cycles involving bats, humans, livestock, and pet animals (Fig. 1). An analysis of the guano of the fruit eating bat *Rousettus leschenaultia* following cultivation of over a thousand bat-associated bacteria showed enrichment for Enterobacteriaceae and putative pathogens; the most cultivated species was *E. coli*, a few strains of which contain known virulence factors [97]. A few bacterial genera containing pathogens pervasive in human and animal diseases (e.g., *Pasteurella, Salmonella, Escherichia*, and *Yersinia* spp.) have been isolated from bats, while other bacterial genera containing pathogens (e.g., *Bartonella, Borrelia*, and *Leptospira* spp.) have novel species unambiguously linked to bat hosts.

### 2.4 Bat-borne pathogen identification

a) Bacterial pathogens in bats

Bat guano mostly reflects the gut bacterial composition, and guano piles have been shown to host potential pathogens. Enteric pathogens from genera such as *Escherichia*, *Enterobacter*, *Yersinia*, *Hafnia*, *Serratia*, *Staphylococcus*, *Streptococcus*, *Pseudomonas*, *Rahnella*, *Micrococcus*, *Acinetobacter*, and *Arthrobacter,* genera containing common zoonotic pathogens including *Bartonella*, *Borrelia*, *Leptospira*, *Campylobacter*, *Clostridium*, and *Bacillus*, unusual Gram-negative pathogens from the genera *Mycoplasma*, *Ureaplasma*, *Rickettsia*, *Anaplasma*, and *Chlamydia*, and confirmed drug resistant pathogens have been reported in several bat studies [51,[97], [98], [99], [100]]. In fact, the diversity of the genera *Chlamydia* and *Mycoplasma* in bats has likely been underestimated [101].

While gut bacteria are obvious candidates for potential zoonotic pathogens, other sites of bats’ bodies may also contain pathogens. The skin microbiomes of bats are highly diverse, and harbour common environmental bacteria such as *Pseudomonas* spp., *Acinetobacter* spp., *Pasteurella* spp. (possibly from the oral cavity via grooming) and Enterobacteriaceae (possibly via faecal contamination of roosts) [102]. Recently it was shown that bat urine contained more microbiota than saliva or faeces, but potential zoonotic pathogens such as *Leptospira*, *Rickettsia*, *Bartonella* and *Coxiella* were found in all body sites [61]. In all these cases, pathogenic potential of the bacteria is yet unknown. However, bat ticks have been confirmed to harbour human pathogens belonging to *Bartonella*, *Borrelia* and *Rickettsia* spp [26,[103], [104], [105], [106], [107]]. A summary of several emerging bat-borne bacterial diseases which may affect humans is shown in Table 3.

i) ***Bartonella* spp.:** Bartonellosis caused by Gram-negative *Bartonella* spp. and spread by the bites of blood-sucking arthropods, is an emerging zoonotic disease [108]. Many animals such as bats, and other wild and domestic mammals harbour *Bartonella* species, including those capable of infecting humans [98,[109], [110], [111]]. Bats seem to be the source for at least two human pathogens, *B. mayotimonensis* and *B. naantaliensis* since they were found in both bat blood and in bat ectoparasites [98]. Later, *Bartonella* genotypes in fruit bats and bat flies biting them were discovered in Madagascar, lending further credence to the possibility of zoonotic transmission to humans [112]. Stuckey et al. reported that the prevalence of *Bartonella* in bats varied from 7.3 % in the Nycteridae to 54.4 % in the Miniopteridae, whereas among the Phyllostomidae, the blood-feeding genera had a higher prevalence about (40 %) than bats with other feeding strategies [113]. Interestingly, in Europe bats strains were highly similar but not identical to clinical strains, but in North America, there was a report of 100 % sequence identity [114].

**Table 3:** A summary of bat-borne bacteria and fungi potentially pathogenic to humans.

Disease	Causative agent (Pathogen type)	Bat species or type	Suggested route of transmission/vector	Other host(s)
Bartonellosis	*Bartonella* spp. (bacterium)	Fruit bats and vampire bats [115]	Bat flies, bat ticks, bat mites	Many wild and domestic animals [98,[109], [110], [111]]
Pasteurellosis	*Pasteurella* spp. (bacterium)	Insectivorous bats [116], big brown bats (*E. fuscus*) [117]	Bat bites or nasal secretions, bat hunting by small predators	Cats, dogs, sheep, and wild birds [116]
Borreliosis or Lyme disease	*Borrelia* spp. (bacterium)	Many bat species worldwide [[118], [119], [120], [121]]	Bat ticks	Rodents and ungulates [122]
Leptospirosis	*Leptospira* spp. (bacterium)	New World Phyllostomidae [[123], [124], [125], [126]]	Bat saliva, urine, faeces (Water contamination)	Rodents and domestic animals [127]
Bloodstream infection	*A. hydrophila* (bacterium)	Vampire bats [128]	Unknown	Fishes, reptiles, and birds [129]
Rickettsiosis	*Rickettsia* spp. (bacterium)	*Pipistrellus* species [130], 17 species in Old and New Worlds [131]	Bat ticks	Dogs, capybaras, cats, and other mammals [[132], [133], [134], [135], [136]]
Typhoid fever	Salmonella typhi (bacterium)	*P. rufus* [137]	Bushmeat of infected bats	Many mammals and reptiles [[138], [139], [140], [141]]
Histoplasmosis	*H. capsulatum* (fungus)	Insectivorous, frugivorous, and nectivorous bats [142,143]	Bat guano	Birds [143]
Coccidioidomycosis or valley fever	*Coccidioides* spp. (fungus)	*Carolliaper spicillata*, *G. soricine* (nectarivore), and *D. rotundus* (vampire bat) [144,145]	Bats and bat guano	Rodents and dogs [[145], [146], [147]]
Candidiasis	*Candida* spp. (fungus)	*G. soricinia* (nectarivore), *M. maior* (tropical house bat), and lesser free-tailed bat (*R. hardwickei hardwickei*) [148,149]	Bushmeat of infected bats, bat guano	Birds and many mammals [149]
Cryptococcal diseases including meningitis	*Cryptococcus* spp. (fungus)	Insectivorous, frugivorous, and nectivorous bats [150,151,152]	Bats and bat guano, tree hollows/trunks visited by bats	Birds and many mammals [150,153]

Constructing a sequence database from over 200 known *Bartonella* strains and 121 cultured strains from bats, McKee et al. proposed that *Bartonella* began infecting mammals 62 million years ago and that the radiation of bacterial clades in this case was tied to the timing of diversification of the mammals [115]. Further, their work suggested that bats are the likely ancestral hosts of all *Bartonella* lineages infecting mammals; bats may have spread the genus around the world and deeply influenced the expansion of this genus to other mammals [115]. Together, these facts support the “bat seeding hypothesis” of evolutionary diversification of potential pathogens. This implies that interaction of bats with other hosts is of importance in the emergence of bat-related zoonoses.

ii) ***Pasteurella* spp.:***Pasteurella* are often commensal microbiota of animals, in the upper respiratory tract, nasopharynx and oral cavity, and are opportunistic pathogens linked to endemic disease and periodic outbreaks [116]. Infections in bats are caused chiefly by *P. multocida,* are often fatal, and may be localized or systemic [51,154]. Although it was thought that the wounds caused by predators, usually domestic cats, were the major route of infections in bats, later it was found that an acute pasteurellosis outbreak caused by *P. multocida* occurred in wild bats not subject to wounding [117]. Whereas transmission to humans may be via bat bites or nasal secretions, so far there is no evidence that is the case. Instead, cats and dogs which commonly cohabit with humans might be the source of most of the human *P. multocida* infections.
iii) ***Borrelia* spp.:** This spirochaete genus contains two known pathogenic groups transmitted by ticks, 1) the Lyme disease causing *Borrelia burgdorferi* and 2] the relapsing fever group (RFG) [155]. Lyme disease is an emerging infectious disease which is spreading to areas not affected when it was first discovered [156], presumably due to range expansion of the ticks transmitting it via climate change [157]. Lyme disease can have serious cardiac and neurological complications in humans [158]. Rodents and ungulates are maintenance hosts for both the tick vectors as well as the *Borrelia* bacteria [122]. The feeding of tick vectors on these hosts as well as bats are likely to be important in disease transmission and persistence. Several novel genotypes have been reported recently in bats from Latin America including tropical regions, which appeared to cluster in bat-specific clades [118,119]. However, known soft tick-associated human pathogens in the Borrelia group, Candidatus *Borrelia johnsonii* and Candidatus *Borrelia fainii*, have respectively been isolated in *Ornithodoros kelleyi* in the United States [120] and *Ornithodoros faini* in Zambia [121]. Many earlier studies reported *Borrelia* prevalence in temperate North America and Europe, but recently, human pathogenic relapsing fever caused by New World strains was detected in bats in Asia [159]. This suggests that humans could play a role in disseminating diseases to bats in the first place via the transport of contaminated material over large distances, and therefore, more studies in this regard are warranted.
iv) ***Leptospira* spp.:** Leptospirosis is the most widespread zoonotic disease in the world, caused by bacteria which colonize the kidneys of both domestic and wild mammals, and is transmitted to humans via water contamination caused by the urine of rodents and possibly bats [127]. Several studies confirmed the presence of *Leptospira* spp. in bats [51,160,161]. Bats may be a reservoir of leptospirosis, even though they are considered unlikely to transmit it directly to humans [[123], [124], [125]]. There is a high diversity of *Leptospira* spp. in bats and asymptomatic infections with relatives of human pathogenic strains seem common [126]. In contrast to *Bartonella* spp. which coevolved with both bats and rodents with negligible switching of hosts, *Leptospira* spp. exhibit a high number of hosts switching events [110]. Therefore, the latter may be able to transmit between bats and rodents more readily, which elevates the possibilities of spill-over to humans [110]. Seasonal events such as bat reproduction might influence transmission rates since they have been linked to simultaneous shifts in the saliva, urinary and faecal microbiomes, which affect the shedding of *Leptospira* spp [162].
v) ***Aeromonas hydrophila*:** Many bats have been shown to harbour this bacterium as part of their normal gut flora [128]. It has been suggested that *A. hydrophila* is necessary especially for vampire bats to digest their blood meals, like leeches [128]. While *A. hydrophila* has been conventionally regarded as a fish pathogen, recent work shows that reptile strains are often multidrug resistant [129]. It is already known that reptile and clinical strains of *Aeromonas* spp. can be identical [163]. *A. hydrophila* and other aeromonads are also found in vegetable matter as well as the excreta of farm animals and humans [164]. They are also reported from rare infections in cattle [165]. However, there is a lack of data on the similarity of bat strains with clinical, veterinary, and environmental strains. Therefore, bats might either be the sources of pathogenic strains or the recipients due to anthropogenic disturbances.
vi) ***Rickettsia* spp.:** The rickettsiae are obligate intracellular parasites and several bacteria belonging to the genera *Rickettsia* and *Orientia* have been flagged as emerging pathogens of public health concern [132]. The major groups of rickettsial pathogens are the typhus group (*R. prowazekii*, *R. typhi*), the spotted fever group (*R. rickettsia*, *R. conorii*, *R. africae*, *R. felis*, *R. australis*, *R. japonica*), and the scrub typhus group (*Orientia tsutsugamushi*, Candidatus *O. chuto*, Candidatus *O. chiloensis*) [[132], [133], [134], [135], [136]]. Common pipistrelle bats *Pipistrellus pipistrellus* in China were found to carry *R. parkeri*, *R. lusitaniae*, *R. slovaca* and *R. raoultii*, among which all except *R. lusitaniae* cause human infections [166]. Bat tick surveys covering other countries of the Old and New Worlds found that they carried *Rickettsia* spp. as well [131], suggesting that ticks might transmit rickettsial diseases between bats, humans, and other animals.
vii) **Enteric pathogens in bats:** Data regarding the normal gut flora of bats and enteric pathogens of bats comes from microbiological surveys over several decades [78,[167], [168], [169], [170], [171]], while some studies also reported on bacterial species that may pose a potential health threat to humans or domestic animals [96,170,171]. Enteric pathogens such as *Salmonella, Shigella, Yersinia*, and *Campylobacter* species have been detected in bats, among which, *Salmonella* is a leading cause of gastroenteritis in both humans and animals such as industrial livestock [[138], [139], [140], [141]]. Investigation of bacterial pathogens in chiropteran species revealed the incidence of *Campylobacter* sp. from the feeding habitats of bats [91,169]. The widespread distribution and host range of entero-pathogenic *Yersinia* spp. (e.g., *Y. enterocoliti*s and *Y. pseudotuberculosis*) pose a potential zoonotic risk of human health. Both bacterial species have continually been isolated from several wild and domestic animals [51]. A high incidence of different *Yersinia* species (35 %) was found in the of insectivorous *Myotis myotis* natural populations in Poland [172], whereby histopathological evaluation revealed inflammatory lesions in several internal organs, purulent meningitis, and interstitial pneumonia.

Non-typhoidal *Salmonella* serotypes have been isolated only rarely from the intestine of bats. Among these, *Anatum, Blockley, Rubislaw, Saintpaul, and Sandiego* are prevalent in farm animals and of interest to human medicine [173], but Salmonella Caracas and S. Llandaff have seldom been linked to human salmonellosis cases [174]. As distinct from broad-host-range *Salmonella* serotypes, *Salmonella typhi* is the causal agent of typhoid fever and associated with human disease [175]. This serotype was isolated in the 1970s from the blood, internal organs, and bile of *Pteropus rufus* from Madagascar [137].

b) Fungal pathogens in bats

Fungi are an increasing threat to human health due to the paucity of approved antifungals, rising resistance to known antifungals, difficulties in diagnosis due to unspecific symptoms and an increased population of immunocompromised individuals. Bats are potential reservoirs for the emerging human pathogens *Paracoccidioides brasiliensis*, *Blastomyces dermatitidis* and *Sporotrichum schenckii* [176]. They could be reservoirs of other pathogenic fungi, but whether they are primary reservoirs is doubtful. An overview of fungi found in bats and potentially pathogenic to humans is shown in Table 3.

i) ***Pseudogymnoascus destructans*:***Pseudogymnoascus destructans* (Pd) [177], formerly named *Geomyces destructans* [177], is the causal agent of White-Nose Syndrome (WNS), a fungal disease of hibernating bats, which spreads rapidly in colonies [178]. There is no known direct threat of *P. destructans* (Pd) exposure to humans and increased arousals from inactivity may increase the recurrence rate at which bats, and humans come into contact. However, it is plausible that bat die-offs may contribute to episodes of increased vector-borne diseases in humans since bats perform the crucial ecosystem function of insect pest control [179].
ii) ***Histoplasma capsulatum*:** This dimorphic spore-forming fungus causes the disease histoplasmosis, which affects the lungs, and the major route of exposure is inhalation of bird or bat guano. It was first isolated from bats in Oklahoma, USA [142] and has a worldwide distribution with eight geographically bounded clades [143]. Pathological analysis following experimental infection in bats revealed that the fungus attacked other internal organs after disseminating from the lung tissue [180]. In some cases, long bone infections were observed [130]. Outbreaks among cave visitors have been reported [181,182]. A multinational outbreak with cases of respiratory failure occurred due to the same reasons in 2013 [183]. The role of bats in disease transmission was confirmed in the 1980s [184]. Later work also showed that histoplasmosis could occur at the entrances of caves, and transmission did not need the subject to enter the interior of the caves [185]. In 2010, a commercially available antigen test for histoplasmosis was modified to detect Latin American clades, while only North American clades could be detected before [186].
iii) ***Coccidioides* spp.:***C. immitis* which is the causative agent of coccidioidomycosis or valley fever, is found in bat guano [144]. Coccidiomycosis is a systemic fungal disease. The US and parts of Latin America are the major regions of occurrence where coccidioidomycosis caused by *C. immitis* and *C. posadasii* are known [[145], [146], [147]]. Bats and bat guano are known sources of these fungi [187], while antibodies against *Coccidiodes* have been characterized in rodents [188], which may be a more important reservoir for coccidiomycosis.
iv) ***Candida* spp.:** Apart from humans, other mammals (cattle, horses, pigs, cats, and dogs) and birds also carry *C. albicans* infections [189]. *C. albicans* has been discovered in the internal organs of bats consumed by humans in Africa [148], highlighting the bush-meat trade particularly the consumption of raw or undercooked bush-meat, as a potential route in the spread of pathogens. *C. albicans* infects the mucous membranes of the mouth and pharynx in humans, while skin and wound infections are also known. In Brazil, studies showed several *Candida* spp. in bat faeces such as *C. guilliermondii*, *C. krusei*, *C. lusitaniae*, *C. parapsilosis*, and *C. pelliculos* [149].
v) ***Cryptococcus* spp.:***C. neoformans* is usually considered a soil fungus and can infect the lungs and the central nervous system of humans and *C. gattii* can cause meningitis [190]. Often animals carrying Cryptococci also carry other potentially pathogenic fungi. Birds and several wild as well as domestic mammals [150] are infected by *Cryptococcus* spp. *H. capsulatum*, *Cryptococcus* spp. and *Paracoccidioides brasiliensis* are known to be shed by insectivorous, frugivorous, and nectivorous bats from urban areas in Brazil [151]. *Cryptococcus* strains including *C. neoformans* were reported from tree hollows and decayed tree trunks in India, a potential reservoir, and an environment likely to be frequented by bats [191]. *Sporothrix schenckii*, *Scopulariopsis* sp. and *C. neoformans* have been reported from bats or bat guano [130,152].

c) Parasitic protozoa in bats

Parasitic diseases could spread due to habitat disturbance of bats leading to dietary and microbiota shifts affecting inter-species transmission [88] and due to vector-borne parasite range expansion via climate change [157,192]. Bats carry a wide range of protozoan parasites, some of which may infect humans [193]. Several possible human pathogens have also been found in bat ticks [26,104]. In a recent survey in Nepal, insectivorous bats were shown to harbour multiple parasitic protozoa of genera such as *Cryptosporidium*, *Eimeria, Entamoeba* and *Giardia* at the same time [194]. Owing to their complex life cycle, climate change driven range expansion of vectors, lack of investment and growing drug resistance, parasites present a challenge for drug development [195]. An overview of parasitic protozoa afflicting bats and pathogenic to humans is shown in Table 4, but data regarding possible transmission of these parasites to humans is not available.

i) ***Babesia* spp.:** Babesiosis infections occur in mammals (including humans) and birds, is usually transmitted via tick bites, and is also increasingly spread through blood transfusion. *Babesia* spp. as well as the related *Cytauxzoon* spp. and *Theileria* spp. belong to the intracellular apicomplexan parasite order Piroplasmida [196]. *Babesia* strains infect the red blood cells and are transmitted by the tick *I. dammini* to humans [197]. Bat ticks carrying *B. venatorum* are known to bite humans [104]. In addition, some bat ticks also carried *Babesia* and *Theileria* species infecting dogs and ruminants [26], although human transmission is not reported.

**Table 4:** A summary of bat-borne protozoan parasites causing human diseases.

Disease	Causative agent	Bat species or type	Suggested route of transmission/vector	Other host(s)
Babesiosis	*Babesia* spp.	*P. rufus*, *M.* cf. *alcathoe*, *M. bechsteinii*, *M. myotis*, *Pi. nathusii*, and *V. murinus* [104,198,199]	Bat ticks	Many mammals [196], Birds [26]
Amoebiasis	*Entamoeba histolytica*	*R. leschenaulti* and *E. spelaea* [194], *R. rex* [200]	Water contamination (Cysts in bat guano?), Insect vectors (cockroaches, flies and coprophagous beetles) [201]	Monkeys [202], Horse, camel, yak, cattle, Tibetan sheep, goat, and Mongolian sheep [203], Pigs [204]
Chagas disease	*Trypanosoma cruzi*	*Noctilio* spp., *Myotis* spp., and *Artibeus* spp [205,206].	Triatominae (biting flies/bugs)	Coati, tamarin monkeys, capuchin monkeys, opossums, rats, armadillos, raccoons, and skunks [[207], [208], [209]]
Malaria	*Plasmodium* spp.	At least 31 species [210,211]	Mosquitoes and other biting insects	Rodents, primates, other mammals, birds, and reptiles [210]
Cryptosporidiosis	*Cryptosporidium* spp.	Straw-coloured fruit bats (*E. helvum*), other fruit bats, and insectivorous bats [[212], [213], [214], [215], [216]]	Bat guano	Dogs, cats, pigs, rats, horses, rabbits, skunks, and chipmunks [217]
Leishmaniasis	*Leishmania* spp.	Free tailed bats, New World leaf-nosed bats, insectivorous bats, and *P. pipistrellus* [[218], [219], [220], [221]]	Phlebotominae (sand flies)	70 species including dogs, rodents, rabbits, sloths, and opossums [222]
Toxoplasmosis	*Toxoplasma* spp.	Silky short tail bat (*C. brevicauda*), free tailed bats, New World leaf-nosed bats, and insectivorous bats [[223], [224], [225], [226], [227], [228]]	Food contamination (Cysts), bat guano or urine	Cats, rodents, pigs, wild boar, and birds [[229], [230], [231]]
Giardiasis	*Giardia* spp.	Insectivorous bats (*R. macrotis*, *R. pusillus*, and *R. pearsonii*) [194]	Water contamination (Cysts in guano?)	Many wild and domestic mammals, and birds [232,233]

**Fig. 2:**
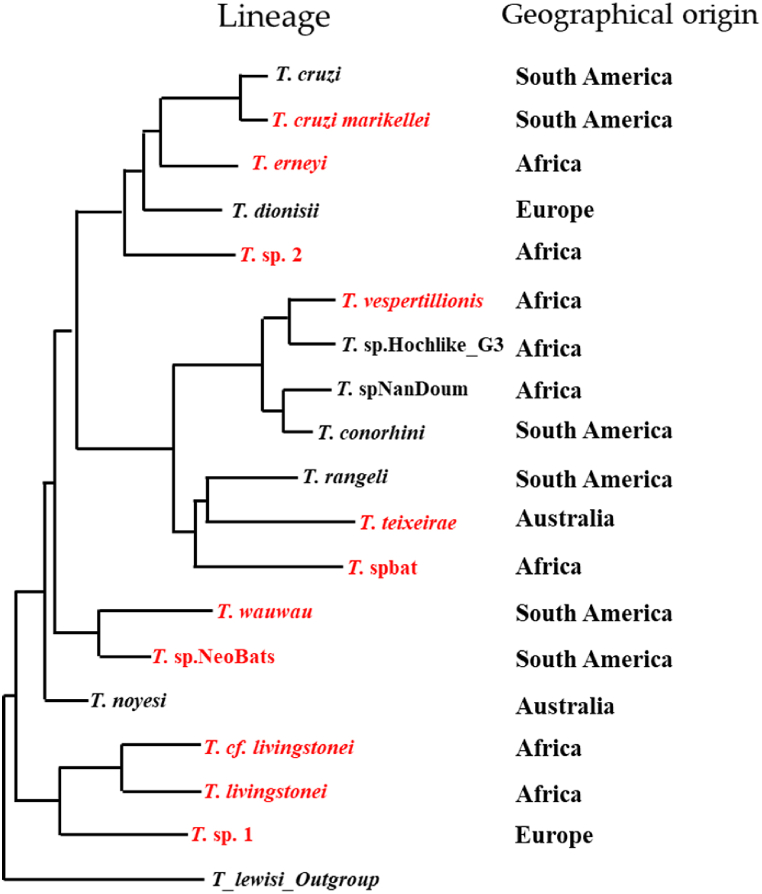
Speciation of Trypanosoma lineages in different hosts showing diversification from bat lineages in Africa. The phylogenetic tree is adapted from Clément et al. [206] and not drawn to scale. The red lineages originate in bats and *T. cruzi* is a major human pathogen.

*B. microti* is a human pathogen with the smallest known apicomplexan genome to date and has highly specialized metabolic adaptations for life inside erythrocytes [234]. Further analysis of the genome revealed that *B. microti* lacks haemoglobin digesting proteases (which explains the failure of the quinine family of antimalarial drugs to cure babesiosis) but contains a 1-deoxy-d-xylulose 5-phosphate reductoisomerase (Dxr) enzyme which makes it susceptible to fosfidomycin [234]. This is important since resistance to antibiotics such as azithromycin and clindamycin is reported [235]. *B. vesperuginis* and *B. microti* have been implicated in bat infections [198,199] suggesting either a possible zoonotic transmission from bats to humans, or a spread to bats due to human activity.

ii) ***Entamoeba* spp.:***Entamoeba histolytica* is a gastrointestinal tract pathogen affecting between 35 and 50 million people annually and causing up to 100,000 deaths/year, whose diagnosis and treatment are complicated [236] It has a high prevalence in areas with poor sanitation [237] and therefore, the control of transmission is considered a better option than treatment [236]. *E. bovis*, *E. moshkovskii*, *E. ecuadoriensis* and *E. histolytica* were found in domestic animals such as horse, camel, yak, cattle, Tibetan sheep, goat and Mongolian sheep in a recent survey in the Tibet-Qinghai region [203], while other studies in China also found pigs infected with several *Entamoeba* species [204] in the southeast, which has a warmer climate. *E. histolytica* has been found in king horseshoe bats *Rhinolophus rex* [200] and this opens the questions about whether they are the source of human infections. It could be also that bats and humans might be exposed to the same common environmental sources of infections, and more data is needed. Dogs, cats, rats, and monkeys can be induced to have experimental infections [238], but natural infections are known from monkeys [202] as well. Further, it has been recently reported that *Entamoeba* spp. harbour CRESS (circular Rep-encoding single-stranded) viruses, some of which are known to infect birds and mammals [239] Cockroaches, flies and coprophagic beetles may transmit the infective cysts between different species including humans, domestic animals, and wildlife [201].
iii) ***Trypanosoma cruzi*:** Chagas disease or American trypanosomiasis caused by *Trypanosoma cruzi* is a bloodstream disease endemic to the Americas transmitted by the *Triatominae* flies. With about 10 million people carrying it, Chagas disease is a re-emergent disease [240]. Long term cardiac, nervous, and gastrointestinal complications can occur [241]. A bat-related *T. cruzi* strain (TcBat) [242] was shown to cause human infection [243]. Wild animals such as coati, tamarin monkeys, capuchin monkeys and opossums in Brazil [207] and urban-adapted animals like rats in Mexico [208] and armadillos, raccoons, opossums, and skunks in the US [209], are known reservoirs. Bats presumably also act as reservoirs by overlapping biotopes with some of these animals, but further details of the transmission cycle need to be worked out. According to the “bat seeding” hypothesis, the *T. cruzi* evolved from an ancestral monophyletic group of bat trypanosomes and switched to other land mammals, initiating further mammal-associated lineages within the same clade [205]. Due to the prevalent trypanosome infections in bats, the discovery of new bat-related *T. cruzi* lineages, multiple trypanosome infections in the *Minoptera* bats, and further molecular taxonomy work, it is now considered that the *T. cruzi* originated as a bat pathogen in Africa and radiated to other parts of the world from there [206] as shown in Fig. 2.

iv) ***Plasmodium* spp.:** Malaria is the major parasitic disease in humans and six strains of *Plasmodium* cause malarial diseases in humans. Rodent strains of malaria (*P. yoelli*, *P. berghei*, etc.) are used as experimental models for the human version of the disease [244]. There is a close relationship between the rodent strains and bat strains of *Plasmodium* spp [210]. Further, species such as arboreal African thicket rats [210] and flying squirrels [211] share habitats with bats and may be bitten by the same insect vectors. So far, there is no evidence that these vectors bite humans or that bat *Plasmodium* spp. infect humans. However, it has been suggested that host-switching from rodents spread malaria to humans [245]. Phylogenetic studies show that bird and reptile-associated *Plasmodium* spp. are not in the same taxon as mammalian ones, and that bats were the likely first mammalian hosts, which spread it to primates and rodents [210], in yet another instance of “bat seeding”.
v) ***Cryptosporidium* and *Eimeria* spp.:** These genera cause gastrointestinal disease in humans and are widely distributed among mammals and birds. *C. hominis*, *C. parvum*, *C. meleagridis*, *C. viatorum*, *C. felis*, and *C. canis* are known from human infections, while *C. muris*, *C. suis*, *C. andersoni*, *C. ubiquitum*, and *C. cuniculus* are also known in humans, with additional genotypes infecting horses, rabbits, skunks, and chipmunks [217]. The major *Cryptosporidium* infecting humans is *C. parvum*, which has been found in bats [212], while bat-associated *C. hominis* has been reported as well [213]. Molecular epidemiological studies led to the conclusion that zoonotic transmission from mice is important for the spread of *C. parvum* infections [214]. Bats have been shown to host *Cryptosporidium* and *Eimeria* spp [194,215,216]. Molecular phylogenetic analysis showed that the bat genotypes V is linked to the clade causing human cryptosporidiosis, suggesting transmissibility to humans [214,215].
vi) ***Leishmania* spp.:** Over 20 species of *Leishmania* parasites cause the complex disease, leishmaniasis, which has three clinically manifested forms – cutaneous, mucocutaneous, and visceral [246]. While the other two forms are disfiguring and debilitating, visceral leishmaniasis is fatal if untreated. The visceral form is caused by *L. infantum* in Europe and the Americas, and in tropical Asia by L. *donovani* [247]. In humans, about 12 million leishmaniasis infections, with up to a million new cases per year, and about 50,000 fatalities annually [222]. Leishmaniasis is spread by the bites of sand flies of the Phlebotominae subfamily from several animal hosts to humans [248]. Dogs and rodents are the main reservoir hosts for *L. infantum*, but bats can also be infected [248]. *Leishmania* spp. have been detected in bats in areas where leishmaniasis is endemic, including urban areas, which further strengthens the reservoir host hypothesis [[218], [219], [249], [250]]. While there have also been reports of bat leishmaniasis where the human disease is not endemic [220], some recent work shows that among all the vertebrates, bats have the greatest degree of spatial overlap with sand fly vectors in the territories occupied, indicating a possibly important role in the transmission cycle [221].
vii) ***Toxoplasma* spp.:** The major pathogen in humans is *T. gondii* with the infection contracted from undercooked food, exposure to the faeces of cats or other animals, and mother to child. It is usually asymptomatic, but eye issues and mild flu-type symptoms may develop, and congenital cases can have eye and neurological complications which may lead to blindness and mental retardation [251]. There have also been documented outbreaks around the world [252]. The distribution and virulence of *T. gondii* is likely to be connected to human impacts such as agricultural expansion into formerly forested areas [253]. Latent toxoplasmosis may affect up to 50 % of the human population and has been linked to other psychological and pathological conditions, constituting a disease burden [254]. Although domestic cats are most associated with human infections, most mammals and birds are potentially afflicted, whereby wild, or domestic swine [[229], [230], [231]] and rodents were shown to be infected [229]. *T. gondii* infections in rodents often makes them fearless and enables them to be predated by cats [255]. Several reports also describe *T. gondii* from bats around the world [[223], [224], [225], [226], [227], [228]]. Considering possible predation by cats and habitat overlap with rodents, bats could be contracting *T. gondii* from these animals, but their role in the transmission cycle is unclear.
viii) ***Giardia* spp.:***Giardia duodenalis* (also called *Giardia intestinalis* or *Giardia lamblia*) is the major giardia parasite in humans, which causes recurrent diarrhoea and is known to be transmitted mainly via contaminated water [256]. Non-human hosts include birds, wild animals, and domestic mammals including dogs, cats, cattle, sheep, and goats [232,233]. Recently, *Giardia* spp. have been discovered in bats in Nepal [194], and work on CRESS viruses suggests that novel viruses can be spread by *Giardia* spp [239]. Given the widespread distribution of *Giardia* spp. in mammals and their discovery in bats, the open question is how transmission occurs between bats and other animals, and whether bats shed *Giardia* parasites along with novel viruses.

d) Viral pathogens in bats

The integration of numerous viruses into the bat genome testifies to tolerance to viral infection over evolutionary history. The presence of bat-specific diversity in microRNAs suggests that regulation of gene expression may also be bat-specific [23]. It has already been mentioned that bats' immunity to viruses may stem from the expression of the APOBEC3 gene [24,25]. It has also been surmised that since the flying lifestyle leads to elevated body temperatures in bats, coevolution with bats predisposes the viruses to resist the innate immune responses of spill-over hosts such as humans [257]. Another hypothesis is that mitochondrial mechanisms enable oxidative stress tolerance during the flight and suppress tumours and pathogens in bats [258].

Many viral pathogens infect bats without manifesting notable symptoms [259], but their pathogenicity is varied. Noticeable exceptions to asymptomatic viral infections in bats include the Rabies and Tacaribe viruses; the latter is a South American Arenavirus that caused pervasive bat mortality in the 1950s [260]. Sonntag et al. linked adenoviruses to bat mortality [261], although such patterns are perhaps predictable given the gap between this gut-infecting DNA virus and other bat-linked (mainly RNA) viruses. Previously, a study confirmed seasonal amplification of RNA viruses, but not DNA viruses, in a monitored insectivorous bat colony in Europe [262]. Hence, different control mechanisms prevail for RNA versus DNA viruses in bats [258]. Most human coronaviruses are descendants of ancestral bat viruses [27]. We consider a few viruses that have caused notable outbreaks since 2003.

i) **Hendra virus (HeV):** Bats became the primary source of zoonotic disease caused by Hendra virus (HeV) in northern Australia in 1994 [263]. In two independent overflow events, this novel paramyxovirus claimed the lives of 15 horses and of two humans who had contact with infected horses [261,264]. A drastic increase in HeV spill-over events in Australia was reported [265]. An outbreak of respiratory illness in pigs and encephalitis in humans led to the first emergence of the Nipah virus (NiV) in Malaysia in 1998, and NiV and HeV were incorporated into a new genus, Henipavirus.
ii) **Nipah virus (NIV):** Another paramyxovirus emerged in Malaysia after the initial eruption of HIV. Dubbed Nipah virus (NiV), it was first isolated from humans, and commercial farm pigs demonstrated respiratory and neurological disease, which was exceedingly contagious [266]. Between September 1998 and April 1999, NiV led to the demise of 105 humans and over 1 million pigs in Malaysia and Singapore. NiV continued to cause regular outbreaks of encephalitis in Bangladesh and India, verifying direct bat-to-human and human-to-human spread and transience of 70–100 % [8]. NiV isolates from *P. vampyrus* varied more than the human and *P. hypomelanus* isolates (98 NT changes) [267]. Serological assessment in the outbreak in Malaysia illustrated that domesticated animals had NiV-specific antibodies [266]. High seropositivity was assessed in 14 species, which showed that two fruit bat species for small flying fox (*Pteropus hypomelanus*) and sizeable flying fox (*P. vampyrus*) had 31 % and 17 % prevalence, respectively. NiV was isolated from *P. hypomelanus* urine and partially eaten fruits, which matched pig and human (56 NT change) isolates [268,269].

Molecular-level results and viral isolation revealed that the fruit bats are the usual reservoir of NiV [270]. Although flying foxes are present in rural and forested areas seasonally; these bats can travel long distances and occasionally roost together in large groups. *Eonycteris spelaea* are nectar-feeding and live in extensive colonies of hundreds, in caves throughout Peninsular Malaysia and a larger area in South and Southeast Asia. Many of these caves are frequently visited by humans. *Scotophilus kuhlii* is insectivorous and found throughout Malaysia and much of Southeast Asia, but roosts gregariously, often using man-made structures. NiV was found in *E. spelaea* urine [270] and antibodies against NiV were found in both *E. spelaea* and *S. kuhlii* [271], but there is no evidence of further transmission.

iii) **SARS coronavirus (SARS-CoV):** SARS-CoV is the causative agent of SARS (Severe Acute Respiratory Syndrome), which was recognized as a novel human disease in China. Principally, palm civets sold in the live animal bazaars were considered the source of SARS-CoV, but other animals such as raccoon dogs were also infected [272]. Three species of horseshoe bats (*Rhinolophus pearsoni, R. macrotis* and *R. pussilus*) demonstrated a moderately high seroprevalence of SARS-neutralizing antibodies. Bat-CoV RNA showed 92 % identity with human SARS-CoV isolates. Another investigation examined nasopharyngeal and anal swabs of 120 bats, where the virus detected matched those found in Chinese horseshoe bats (*R. sinicu*s). It showed 88 % identity SARS-CoV with an immense variation in spike genes [273]. Bat SARS-CoV-like viruses are phylogenetically distinct from human SARS-CoV and hence not intimately related. Therefore, bat SARS-CoV cellular receptors could not bind to human angiotensin transfer enzyme II (ACE2) to penetrate the cell [274]. The receptor-binding domain of the spike protein was isolated from live SARS-like CoV in bat faecal samples. Ge et al. [2013] established *in-vitro* that this virus had a unique species tropism and could utilize human, Chinese horseshoe bat and civet ACE2 receptors for cell entry [275]. Additionally, changes in land use and the presence of livestock have been recognized as triggers for the transmission of SARS-related coronaviruses [276].
iv) **MERS coronavirus (MERS-CoV):** In 2012, a new coronavirus was isolated from a man with mild pneumonia and subsequent renal failure in Saudi Arabia; this was the Middle East Respiratory Syndrome coronavirus or MERS-CoV [277]. It transmitted from camels to humans and this hypothesis is supported by a complete genome sequence [278]. MERS-CoV antibodies and RNA have also been detected in camels during the epidemic [279]. Phylogenetic analysis showed that MERS-CoV fits into lineage C of the genus Beta-coronavirus alongside bat-CoV viruses HKU4 and HKU5 [280]. Thus, bat viruses are ancestors of MERS-CoV and camels act as reservoir hosts that spread it to humans. The diversity of MERS-related CoVs in bats has been explored in Saudi Arabia [281], Africa [282,283], Europe [283], and eastern Asia [284]. Data on receptor usage by MERS-CoV hints at bats as the source of MERS. Subsequently, di-peptidyl peptidase 4 (DPP4 or CD26) was recognized as the cellular receptor for MERS-CoV [285]. Bat coronavirus HKU4 is closely related to MERS-CoV and can use DPP4 as a receptor to commence cellular entry. However, although bats and alpacas may be potential reservoirs for MERS-CoV, camels were held responsible for the spill-over to humans [286].
v) **Filoviruses:** Ebola and Marburg, amongst the most dangerous viruses that infect humans, are filoviruses. Regardless of their impact, the reservoir for these viruses was not known definitively. Viral RNA specific to both Ebola and Marburg has been identified in various fruit bat species in Gabon and the Democratic Republic of Congo [287,288]. The incidence of Marburg haemorrhagic fever in mine employees in southern Uganda was ascribed to feasible transmission from infected bats (*Rousettus aegyptiacus*) that had colonized the mine. Genetic analysis proved that the Marburg virus isolated from the contaminated mine employees was very similar to those in the *R. aegyptiacus* [289]. Later it was shown that *R. aegypticus* shed the Marburg virus upon experimental infection [290].
vi) **COVID-19 (SARS-CoV-2):** This coronavirus is from the family Coronaviridae, order Nidovirales. Coronaviruses (CoV) circulating in bats include many relatives of SARS-CoV-2, but SARS-CoV-2 itself was not found in bats. Numerous and widely present, *Rhinolophus* bat species in Europe carry viruses related to SARS-CoV-2 [291], while bats and pangolins in southeast Asia also have similar circulating viruses [292]. Topical studies indicate that more than 500 CoVs have been identified in bats in China, with most living near human populations [[293], [294], [295], [296]]. However, the transmission route to humans at the start of this pandemic event remains unclear. A recent analysis of the Sarbeco virus lineage, which contains SARS and SARS-CoV-2, showed that those two branches undergo frequent recombination [297]. By 2014, around 200 novel coronaviruses had been accredited in bats, and about 35 % of the bat virome sequenced to date is of coronaviruses [298]. Nonetheless, SARS-CoV-2 itself is not a product of such a recombination event; it shares the receptor-binding domain crucial to ACE-2 binding in humans with ancestral bat lineages and has been given a divergence date of 1948 [297].

Bats are hunted and sold to restaurants for food; they are atypical in Chinese markets but more common in Southeast Asia. A recent study examined the animals sold in Wuhan wet markets before the pandemic and concluded that bats and pangolins were unlikely to be the spill-over hosts [299]. The current hypothesis is that an intermediary host animal plays a role in the transmission. Some suggest that rodents such as marmots might be the likely candidates since they can contract the virus but do not usually die [300]. To avert future outbreaks like the current pandemic, strengthening food control and market hygiene activities in the live food market will be essential to protect people from zoonotic diseases [301].

### 2.5 Routes of transmission of bat-borne diseases to humans

Joffrin et al. proposed four significant ways for the bat zoonotic cycle to connect to humans: intermediate hosts, blood-feeding arthropods, bat body fluids, and environmental transmission [302]. In some cases, both intermediate hosts and arthropod vectors are implicated.

a) **Bat bites and body fluids:** Bat bites may be the core communication route for some diseases. Transmission through bat bites has been proposed mainly for the rabies virus (*Rhabdoviridae*). The common vampire bat (*D. rotundus*) can transmit rabies during biting to feed on blood, particularly livestock, and occasionally on humans [303]. Moreover*,* bacteria belonging to *Mycoplasma* spp. have been identified in common vampire bat blood and saliva and can transmit between bats using aggressive behaviours [304]. Obligate blood-feeding bats are restricted to Central and South America with minimal species diversity (<0.005 %). The putative first human case of the 2007 Ebola epidemic in the Democratic Republic of Congo is suspected of having bought bats for consumption [305]. The fruit bat *Eidolon helvum* was commonly hunted and traded bat species in numerous African countries, with more than 120,000 *E. helvum* sold annually [306]. Contaminated bat meat and blood may be to blame for at least some infections since henipaviruses were detected in *E. helvum* bushmeat in the Republic of Congo [307].

Generally, bat species do not bite humans unless deliberate contacts arise (e.g., veterinarians and field biologists involved in bat capture and handling, people annoying bats to eliminate them from homes). Therefore, rather than bites, contact with bat body fluids (saliva, urine, blood) or excreta is a principal mechanism of pathogen transmission to humans. Nipah virus (*Paramyxoviridae*) human illness cases reported in Bangladesh were correlated with the use of raw sap from date palm trees infected with fruit bat saliva and urine [308]. This was further supported by the fact that bats rarely contacted the sap on trees where the collection pots were covered [309]. Therefore, these interventions could dramatically reduce NiV spill-over to humans. In the Marburg virus (Filoviridae) case, investigations pointed out that inter-species transmission may arise via saliva and aerosols, like other hosts by an equivalent mechanism. The observation supports this theory that humans who contracted the Marburg virus had entered bat (*R. aegyptiacus*) caves before falling sick and had regular contact with bats and their bodily secretions [310].

b) **Insect and arachnids (arthropod vectors):** Bats are known to be parasitized by many types of ectoparasites such as insects (bugs, fleas, and bat flies) [311,312] and arachnids (ticks and mites) [313,314]. Cicimid bugs transmit *Trypanosoma* spp [315]. and may bite humans. Bat flies, ticks, and mites all carry *Bartonella* bacteria which could infect humans [316,317], but direct proof of transmission to humans is lacking. Modern studies have overturned the notion that bat flies are generic feeders, and bat flies are now considered highly host-specific [318]. However, an old report [319] and anecdotal observations suggest bat flies occasionally bite humans. More data is needed on bat fly-human interactions to determine their role in zoonotic transmission.

Blood-sucking arachnid vectors such as ticks are essential in connecting bats to other mammals (and possibly birds and reptiles) in disease transmission cycles. Ticks transmit bacteria, protozoa, and viruses to wild and domestic animals and the human population. Hard ticks of the Ixodidae family are the chief vectors in temperate regions with five known bat-exclusive species [[320], [321], [322]]. In contrast, soft ticks of the Argasidae are dominant in the subtropical and tropical areas of the world with over 70 species specialized in feeding on bats [323]. The castor bean tick *I. ricinus* needs up to three different hosts to complete its lifecycle. The larvae feed on insectivores of the Eulipotyphla order (hedgehogs and gymnures) but may also attack rabbits, rodents, reptiles, birds, or bats. The adults attack larger mammals such as sheep, cattle, horses, deer, dogs, and humans, while the immature stages (nymphs) feed on smaller mammals [324]. Recently, the bat tick *Ixodes simplex* was found colonizing humans in Europe [325], while human infestation by *I. vespertilionis* is also known [326]. *Amblyomma* spp. Hard ticks feed on large domestic and wild animals such as horses and capybaras [327,328], but *A. sculptum* transmits the bacterium *R. rickettsia*, which is the causative agent of Rocky Mountain spotted fever, was reported from bats in Brazil [329].

A meta-analysis of hard ticks from the Neotropical zone covering Latin America and the Caribbean reported that no tick species was confined to a single host species, and most could parasitize hosts belonging to different families or orders [330]. In addition, the same analysis suggested that host specificity is lower for immature ticks compared to adults and that the ecological similarity of the hosts was more critical than host phylogeny for the tick-host relationship [330]. It is estimated that out of 700 hard tick species, only five are bat-exclusive, and of 193 presumed soft tick species, 70 specialize in bat feeding. Therefore, it may be sufficient for bats to share a habitat with other animals to allow bat ticks access and transmit bat-borne diseases. Further, the restrictions imposed by ecological specialization may be overcome by interactions of the host with predators, for example [331]. There is insufficient knowledge on the consequences of predation events of bats by other animals (monkeys, domestic cats, etc.) for the transmission of infectious agents.

Ticks can modulate the expression of a core set of proteins in the total proteome, which enables them to feed on different hosts [332]. Gene duplications are known to expand resource utilization in other types of animals [333], allowing the evolution of host shifts. Ticks live in open areas, forests, and meadows, unattached to their host for about 90 % of their lifetimes [334], increasing the possibility of random attachment to different hosts. In addition, ticks exhibit low rates of active dispersal, relying on host movement for dispersal [335] and long generation times of 1–6 years [313]. Since bats are both wide-ranging and long-lived species, bat ticks may be ideal vectors to spread pathogens affecting bats to other animals.

c) **Intermediate animal hosts:** Interestingly, even though they can transmit several diseases to humans, bats are not direct relatives of the primates and rodents (which are Euarchontoglires or Supraprimates) but, according to molecular phylogenomic analysis, belong to the Laurasiatheria clade and are a sister group to the Ferungulata (pangolins, carnivores, and ungulates) [336]. The connections between bat diseases and rodents need closer examination since rodents are already implicated in over 60 zoonotic diseases [337,338] and may overlap with bats' ecological preferences. Recently, coronaviruses related to SARS-CoV-2 (causing the current global pandemic) have been circulating in bats and pangolins in Southeast Asia [292]. Thus, it is likely that livestock, wild ungulates, and carnivores are intermediate hosts for spreading many diseases of bat origin to humans.

Wild animals such as apes, monkeys, and antelopes with bat-borne infectious agents also play a role in the communication chain with humans, such as the Ebola virus. In the case of the severe acute respiratory syndrome (SARS) coronavirus, it is possible that civets *(Paguma larvata*) got contaminated with a virus circulating in horseshoe bats (*Rhinolophus* sp.) and acted as a transitional amplifying host [339,340]. Rather than wild animals, the role of farm animals as midway and amplifying hosts among wild animals and humans has been confirmed in several outbreaks like those of filovirus and henipavirus [341]. The growth of mercantile pig farms in Malaysia with fruit trees on the farm has formed an environment where bats could drop partially eaten fruits contaminated with the Nipah virus into pig stalls [342].

A long-term and asymptomatic spread of viruses from livestock before transmission to humans was suspected to be responsible for an outbreak of the Middle East respiratory syndrome (MERS). Even though bats are expected to be a source of MERS-like coronaviruses, dromedary camels (*Camelus dromedaries*) act as the standard reservoir in which the MERS coronavirus has persisted for a long duration before its initial recognition in humans [278]. Other animals such as llamas (*Lama glama*) and wild boars (*Sus scrofa*) have a recognized vulnerability to MERS coronavirus infection. The human MERS coronavirus was a progeny of camelid-associated viruses, showing that domestic animals play the primary role in the long-time persistence and ultimate transmission of zoonotic viruses to humans.

d) **Environmental transmission:** Transmission from the environment may occur by two mechanisms in the ecosystem, direct and indirect. Of these, indirect transmission to humans by the intermediate hosts such as domestic mammals (cats, dogs, cows, horses, pigs, donkeys, camels, etc.), wild mammals (monkeys, deer, etc.), domestic birds (poultry, ducks, turkeys, etc.), wild birds (crows, sparrows, pigeons, migrant birds, etc.), rodents and ectoparasites (mosquitoes, fleas, ticks, etc.). Indirect transmission may also occur by bat guano (i.e., accumulation of bat excrement in the environment), as shown in Fig. 1.

Direct transmission via bat body fluids (saliva, urine, and faeces) is progressively recognized as an essential mechanism of infecting humans. Human encroachment into bat habitats and increasing urbanization facilitate bat roosting in artificial structures, doubtless increasing contact with bat excreta or body fluids. For example, Nipah virus (Paramyxoviridae) human infection cases were related to the consumption of raw sap from *Phoenix dactylifera* trees contaminated with *P. giganteus* fruit bats’ saliva and urine [308]. In the case of the Marburg virus (Filoviridae), experimental studies indicate that bat-to-bat transmission could occur via saliva and aerosols, suggesting that the virus could also be transmitted to alternative hosts by an identical mechanism [289,290]. This hypothesis is supported by investigations revealing that almost all humans infected with filovirus had entered bat (*R. aegyptiacus*) caves before becoming ill and had regular contact with bats or their secretions [343].

### 2.6 Anthropogenic effects on bat zoonoses

**Fig. 3:**
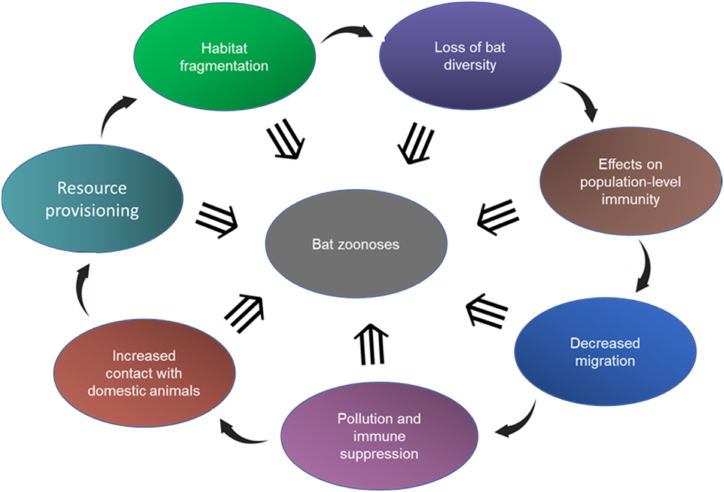
Anthropogenic factors affecting zoonotic disease emergence in bats.

The recent rise in contagious diseases associated with bats sparked a global interest in bats as potential reservoir hosts and vectors of zoonotic pathogens [17,51,344,345]. Bats are among several groups of animals, including birds, foxes, monkeys, raccoons, and rodents considered synanthropic (urban-adapted) [346,347]. Many factors such as bat species richness, habitat fragmentation, the presence of livestock and predatory pets (cats, dogs, and others), changes in food availability, pollution, loss of bat diversity, and population changes are thought to alter the bat zoonotic transmission cycles (Fig. 3). Recent studies show that among mammalian and bird hosts, the capacity to carry zoonotic viruses is homogenous among taxonomic orders, and bats as a taxonomic order only take more human-infecting viruses because of their high species richness [348,349].

Anthropogenic activities heighten the risk for potential zoonotic transmission in urban and semi-urban areas [350]. These risks increase partly because interspersing natural areas with farmland and housing (increases in “ecotonal areas”) alters the ecological niches of pathogens by bringing together humans, vectors, domestic animals (including pets and livestock), and wildlife [351]. The fragmentation of forest habitats was shown to increase the transmission of bacterial zoonoses among humans, primates, and livestock in Uganda [352]. Such an increased transmission effect due to the confluence of humans, livestock, and bats are well-documented for Nipah virus zoonoses in Malaysia [353]. In this case, the intensification of agriculture and the introduction of pigs in areas populated by flying foxes played a role in the emergence and priming for the persistence of zoonosis [342].

Livestock introduction leading to changes in connectivity between bat populations may also play a role in zoonosis. During Hendra virus outbreaks, urbanized flying foxes become more prone to infections due to a decline in migratory behaviour and contact with other flying fox colonies, leading to waning population-level immunity [354]. Human activity can also alter the food supply of bats (resource provisioning), affecting bats' population density. The decline in natural food sources and increased flowering plants in urban areas in Australia led to concentrations of flying foxes. They played a part in the emergence of the Hendra virus in horses and people [354]. Mechanistic models for wildlife-pathogen interfaces suggest that, unless anthropogenic food sources can improve the immunity of reservoir hosts or reduce diet-based pathogen exposure, increases in host population density will significantly increase the chances of pathogen invasions long-term persistence [355,356].

The stresses of pollution, non-native species, noise, and artificial illumination are detrimental to bat health in the urban environment [357], increasing susceptibility to vector-borne infections. The presence of heavy metals and pesticides can suppress immunological defences in humans and many other animals [358]. In species-diverse communities, reservoir species such as bats are predicted by simulations to experience a dilution effect in disease susceptibility [359]. In contrast, extinctions of bats or other losses in diversity in generally species-poor urban areas will have the opposite effect, increasing zoonoses in bat reservoirs. Urban areas could be acting as “ecological traps,” which are attractive but lower quality habitats compared to more natural ones (such as forests), leading to impaired bat health [360]. Therefore, it could be the effects of stressful living in urban areas that are causing the emergence of diseases in bats, which may spill over to humans, rather than bats being *de facto* sources of human pathogens.

### 2.7 Future directions

Industrially beneficial bacteria may be derived from bat species for the biomedical, agriculture, fertilizer, and food fields. Bats are essential in studying emerging bacterial, fungal, and protozoan parasitic diseases. More investigations into containment measures are needed to restrict the possibility of spill-over of novel viruses to humans. More studies into the connections between pathogen lineages in bats and other hosts are also warranted, considering the “bat seeding” aspect of pathogen evolution. Climate change, habitat destruction, hunting, and human population expansion have brought zoonotic diseases to new areas not previously exposed to these threats [28,[361], [362], [363], [364], [365], [366]].

Global database-derived modelling studies suggest that forested regions undergoing rapid changes in land use and harbouring high mammal species richness are possible hotspots of disease emergence [367]. Jones et al. developed a model for Nairobi, which appears to have broad applicability for any large urban area in the tropical or subtropical regions of developing countries with a high population. They identified the types of places where humans, domestic and wild animals converge and zoonotic diseases could emerge [361]. They identified three types of urban environments that are essential for zoonotic transmission - ecotonal interfaces (habitat edges such as borders between cities and forests/grasslands), evolving landscapes (informal settlements possibly involving backyard farms and animals), and managed landscapes suitable for wildlife (parks, gardens, small sanctuaries, etc.) [361]. Therefore, studying bats in these environments will be crucial to understanding bat zoonoses.

Increasing interest in bat-borne viruses and the availability of molecular biotechnology have made studies of new viruses more common globally. Until now, viruses detected in bats are classified under 22 families, several of which are novel (http://www.mgc.ac.cn/DBatVir/) [298]. Viral metagenomics may afford an overview of viral bat ecology and enable the detection of emerging human pathogens [368]. Particularly, assessing the microbes associated with bat flies [369] or bat ticks [131], might be an important way to track potential zoonotic transmission from bats [369], as this allows infectious agents in bat blood to be assessed non-invasively. Molecular methods are becoming more critical to track bat zoonoses, for example, to assess the risks of bat virus transmission [370,371]. Especially metagenomic analysis of bat microbiomes in comparison with strains from other species and the documentation of microbiome shifts in bats can provide critical clues about zoonotic transmission.

Letko et al. have proposed two more essential tools for the study of bat-related zoonoses – the use of bats as animal models (*in vivo*) and the use of human cellular receptors to test viruses obtained from bat studies (*in silico* and *in vitro*), to determine transmissibility to humans and domestic animals [372]. More studies are urgently needed to track transmission cycles for suspected human pathogens in the field and to determine the host preferences and biting behaviours concerning humans, bat flies, and other ectoparasites. More investigations into containment measures are needed to restrict the possibility of spill-over of novel viruses to humans. In the case of identified human pathogens, experimental infections of bat-derived microorganisms in carnivores, ungulates, and rodents in the lab may offer vital clues on transmission.

## 3 Conclusions

To satisfy the growing demand for sustainable development of agriculture, bio-medical, fertilizer, and food supplies the exploration of natural products from microbiomes of bats and other wild animals will be useful. Bats were projected as the natural reservoirs of pathogens causing severe human diseases. However, the role of bats in infectious diseases needs to be further scrutinized. The lack of direct experimental data on transmission from bats to intermediate animal hosts (which transmit to humans) needs to be addressed. In addition, more routine use of One-Health-based ecosystem perspectives considering wildlife studies, disease ecology, and clinical disease will be necessary to tackle future contagions. The Covid-19 pandemic and climate change issues have also underlined the importance of international cooperation and data exchange in combating current and emerging diseases.

## Author contributions

All authors wrote this review paper. J. D. and K.K. conceived the contents. A.P improved the content. B.K. summarized the contents. G.N.P. supervised and finalized the overall content.

## Funding and acknowledgments

We thank the JSPS Invitational fellowship for A.P, Uehara memorial foundation and JSPS Kakenhi Grants in Aid for Scientific Research (23H02607) and Challenging exploratory grant (22K19291) to G.N.P.

## Declaration of competing interest

The authors declare that they have no known competing financial interests or personal relationships that could have appeared to influence the work reported in this paper.
